# Agonist and antagonist properties of an insect GABA-gated chloride channel (RDL) are influenced by heterologous expression conditions

**DOI:** 10.1371/journal.pone.0254251

**Published:** 2021-07-07

**Authors:** Charles L. C. Smelt, Victoria R. Sanders, Alin M. Puinean, Stuart J. Lansdell, Jim Goodchild, Neil S. Millar

**Affiliations:** 1 Division of Biosciences, University College London, London, United Kingdom; 2 Syngenta, Jealotts Hill International Research Centre, Bracknell, Berkshire, United Kingdom; Weizmann Institute of Science, ISRAEL

## Abstract

Pentameric ligand-gated ion channels (pLGICs) activated by the inhibitory neurotransmitter γ-aminobutyric acid (GABA) are expressed widely in both vertebrate and invertebrate species. One of the best characterised insect GABA-gated chloride channels is RDL, an abbreviation of ‘resistance to dieldrin’, that was originally identified by genetic screening in *Drosophila melanogaster*. Here we have cloned the analogous gene from the bumblebee *Bombus terrestris audax* (BtRDL) and examined its pharmacological properties by functional expression in Xenopus oocytes. Somewhat unexpectedly, the sensitivity of BtRDL to GABA, as measured by its apparent affinity (EC_50_), was influenced by heterologous expression conditions. This phenomenon was observed in response to alterations in the amount of cRNA injected; the length of time that oocytes were incubated before functional analysis; and by the presence or absence of a 3’ untranslated region. In contrast, similar changes in expression conditions were not associated with changes in apparent affinity with RDL cloned from *D*. *melanogaster* (DmRDL). Changes in apparent affinity with BtRDL were also observed following co-expression of a chaperone protein (NACHO). Similar changes in apparent affinity were observed with an allosteric agonist (propofol) and a non-competitive antagonist (picrotoxinin), indicating that expression-depended changes are not restricted to the orthosteric agonist binding site. Interestingly, instances of expression-dependent changes in apparent affinity have been reported previously for vertebrate glycine receptors, which are also members of the pLGIC super-family. Our observations with BtRDL are consistent with previous data obtained with vertebrate glycine receptors and indicates that agonist and antagonist apparent affinity can be influenced by the level of functional expression in a variety of pLGICs.

## Introduction

Pentameric ligand-gated ion channels (pLGICs) are a diverse family of neurotransmitter receptors found in both vertebrate and invertebrate species [1, 2]. They include inhibitory receptors, such as the γ-aminobutyric acid (GABA)-gated chloride channels and excitatory receptors such as nicotinic acetylcholine receptors (nAChRs). In insects, GABA-gated chloride channels are targets for a range of insecticides including cyclodienes (e.g. dieldrin), phenylpyrazoles (e.g. fipronil) and isoxazolines (e.g. fluralaner) [3, 4]. The insect GABA receptor that has been studied most intensively is RDL (an abbreviation of ‘resistance to dieldrin’), which was originally identified in *Drosophila melanogaster* by genetic screening for a mutation associated with insecticide resistance [5]. Subsequently, additional resistance-associated target site mutations have been identified in RDL from a variety of species [3, 6, 7].

The functional properties of RDL have been examined extensively in a variety of heterologous expression systems [8–11]. In common with other pLGICs, RDL is an oligomeric protein in which five subunits co-assemble to form a transmembrane ion channel [12]. The binding site for GABA (the orthosteric agonist binding site) is located in the extracellular domain at the interface of adjoining subunits [13]. GABA-gated chloride channels can also be modulated by a variety of allosteric ligands that bind to locations other than the orthosteric site and which can either potentiate or inhibit responses to GABA [14]. In addition, there is evidence that, in the absence of an orthosteric agonist, GABA-gated chloride channels can be directly activated by compounds such as propofol that bind to an allosteric site [15, 16]. Such compounds have been described as allosteric agonists and have been identified for a variety of pLGICs [15, 17–20].

In the present study we have cloned RDL from the bumblebee *Bombus terrestris audax* (BtRDL) and examined its pharmacological properties by functional expression in Xenopus oocytes. An unexpected observation was that agonist EC_50_ and antagonist IC_50_ values determined with BtRDL were influenced markedly (up to ~17-fold) by differing expression conditions. In addition to changes in sensitivity to the orthosteric agonist GABA, similar expression-dependent changes were observed in sensitivity to an allosteric agonist (propofol) and also with a non-competitive antagonist picrotoxinin. The present findings concerning an insect GABA-gated chloride channel are consistent with the evidence of expression-dependent changes in apparent affinity that have been reported for vertebrate glycine receptors and other unrelated receptors and channels.

## Materials and methods

### Plasmids and reagents

Plasmid pGEMHE-DmRDL, containing the *Drosophila melanogaster* RDL cDNA splice variant 3a/6c cloned downstream of a T7 promoter [21] was provided by Sarah Lummis, University of Cambridge, UK. Plasmid pTB207-CeRIC3 (also known as pTB215), containing the *Caenorhabditis elegans* RIC-3 cDNA cloned downstream of a T7 promoter [22] was provided by Thomas Boulin, École normale supérieure, France. All compounds were purchased from Sigma-Aldrich.

### Molecular cloning of BtRDL and DmNACHO

Bumblebee (*Bombus terrestris audax*) colonies were obtained from Biobest (Westerlo, Belgium). Total RNA was extracted from bumblebee heads using TRIzol reagent (Invitrogen Life Technologies). First strand cDNA synthesis was performed with SuperScriptIII reverse transcriptase (Invitrogen Life Technologies) and oligo(dT) primers. BtRDL cDNA (GenBank accession number MZ169389) was amplified by polymerase chain reaction (PCR) using forward and reverse oligonucleotide primers (5’-GAG ACG CGG ACG GCA CGC AGA GA-3’ and 5’-CGT TTC GTCCATCGT ACA CGC GC-3’, respectively) and reamplified with internal forward and reverse oligonucleotide primers (5’-ACG CAG AGA GGC GCC GGG TCA AC-3’ and 5’-CGT TCT CTT CGT TTA TTT CGC CT-3’, respectively). The PCR product was purified with QIAQuik PCR purification kit (Qiagen) and ligated into plasmid pCR2.1 (Invitrogen). BtRDL cDNA was sub-cloned from pCR2.1 into the *Eco*RI site of plasmid expression vectors pSP64GL and pGEMHE. A cDNA clone encoding the *Drosophila melanogaster* chaperone protein DmNACHO (GenBank accession number MZ169390) was isolated from a Drosophila head λZAPII cDNA library (provided by Ron Davis, Baylor College of Medicine, USA) using forward and reverse oligonucleotide primers (5’- TGT TTT GGT AGG GTG TTG TAT TGG CTA GGG-3’ and 5’- CAT CTC GAG CCC AAC TTA AGG TGC TAA AAC -3’, respectively). DmNACHO cDNA was sub-cloned into the *Eco*RI site of plasmid expression vector pSP64GL. The amino acid sequence was identical to the *D*. *melanogaster* predicted protein Dmel_CG13920 (see GenBank accession NP_001286902.1). It is also identical to the protein encoded by the Drosophila gene (NM_001299972) identified by Gu et al. [23]. The amino acid sequence of DmNACHO isolated in the present study differs by 23 amino acid from that presented in figure S2 of Gu et al. [23], however we have been informed that this is due to an incorrect sequence having been used for figure S2 of the 2016 paper (personal communication, Shenyan Gu).

### RNA synthesis and oocyte expression

Plasmid expression vectors were linearised by restriction enzyme digestion at sites downstream from the inserted cDNA. Plasmid pGEMHE-BtRDL was linearised with either *Nhe*I, to retain the Xenopus β-globin 3’UTR [pGEMHE-BtRDL(+3’UTR)], or with *Xba*I, to remove the Xenopus β-globin 3’UTR [pGEMHE-BtRDL(-3’UTR)]. Plasmid pSP64GL-BtRDL was linearised with either *Bam*HI, to retain the Xenopus β-globin 3’UTR [pSP64GL- BtRDL(+3’UTR)], or with *Not*I, to remove the Xenopus β-globin 3’UTR [pSP64GL- BtRDL(+3’UTR)]. All other plasmid constructs were linearised with enzymes that retained the Xenopus β-globin 3’UTR (*Bam*HI for pSP64GL-DmNACHO; *Nhe*I for pGEMHE-DmRDL and pTB207-CeRIC-3). Linearised plasmids were purified with QIAQuik PCR purification kit (Qiagen) and transcription of cRNA was carried out using mMESSAGE mMACHINE SP6 and T7 kits (Ambion, Life Technologies). Oocytes were injected with either 0.97 ng (32.2 nl of 30 ng/μl) or 9.66 ng (32.2 nl of 300 ng/μl) cRNA using a Drummond variable volume microinjector. For convenience, these amounts of cRNA are referred to subsequently as 1 ng and 10 ng.

### Oocyte electrophysiology

Adult female *Xenopus laevis* frogs were obtained from the European Xenopus Resource Centre at the University of Portsmouth. Animals were sacrificed using Schedule 1 procedures approved by the Animals (Scientific Procedures) Act 1986 and by the UCL Research Ethics Committee. Xenopus were anaesthetised by immersion in 0.2% MS222 for 15 minutes (or until complete anaesthesia was confirmed by absence of leg-withdrawal and righting reflex), followed by cranial concussion, decapitation and pithing. Xenopus oocytes were isolated, maintained and injected with cRNA, as described previously [24]. Two-electrode voltage-clamp recordings were performed using a Warner Instruments OC-725C amplifier (Harvard Apparatus) with the oocyte membrane potential held at -60 mV, as described previously [25]. Oocytes were continuously perfused with a modified Ringer’s solution (115 mM NaCl, 2.5 mM KCl, 1.8 mM BaCl2, and 10 mM HEPES, pH 7.3). Application of compounds was controlled by LabChart software (AD Instruments) using a BPS-8 solenoid valve solution exchange system (ALA Scientific Inc). Typically, agonists were applied for 5 s or until a plateau in the response was observed. Antagonists were pre-applied for 30 s and then co-applied with agonist for 5s or until a plateau in the response. was observed.

### Statistical analysis

For individual pairwise comparisons, statistical significance was determined using unpaired Student’s t-tests.

## Results

### Molecular cloning of BtRDL

**Fig 1:**
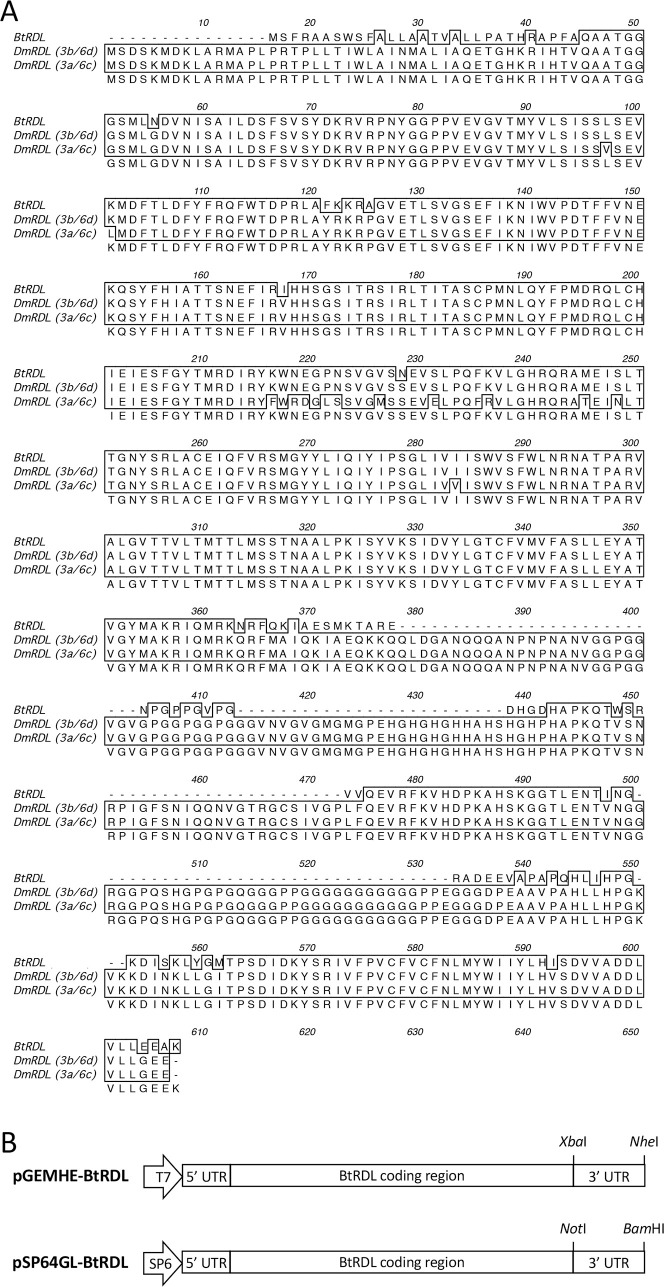
*Bombus terrestris* RDL (BtRDL) amino acid sequence and plasmid constructs. A) Alignment of the predicted amino acid sequence of BtRDL (cloned in the present study) compared with that of two alternatively spliced isoforms of DmRDL (3a/6c and 3b/6d, corresponding to NCBI database accession numbers NP_729461.1 and NP_001261616.1, respectively). The regions corresponding to exons 3 and 6 are indicated below the amino acid sequences, as are the positions of the transmembrane regions M1-M4. B) Representation (not to scale) of the cDNA insert region of plasmid expression vectors pGEMHE-BtRDL and pSP6GL-BtRDL, illustrating two alternative sites at which plasmids were linearised by restriction enzyme digestion to either remove or retain the Xenopus β-globin 3’ UTR region (*Xba*I and *Nhe*I, respectively, for pGEMHE-BtRDL and *Not*I and *Bam*HI, respectively, for pSP6GL-BtRDL). Also indicated are the upstream promoters (T7 or SP6) of the two plasmids.

A full-length cDNA clone of *Bombus terrestris* RDL (BtRDL) was isolated from total RNA prepared from *B*. *terrestris* heads. Nucleotide sequencing identified an open reading frame of 483 amino acids and indicated that the BtRDL cDNA isolated in this study corresponds to what has been described in other insect species as the RDL 3b/6d splice variant [26, 27]. A comparison of the BtRDL mature protein (i.e., the protein sequence excluding the cleaved N-terminal signal peptide) with that of the *Drosophila melanogaster* RDL (DmRDL) slice variant 3b/6d revealed 72% amino acid identity (Fig 1A). However, the two receptors were found to share a much greater level of amino acid identity (97%) if the variable intracellular loop domain, located between transmembrane domains M3 and M4, was excluded from the comparison.

### Influence of 3’ UTR on GABA EC_50_

**Fig 2:**
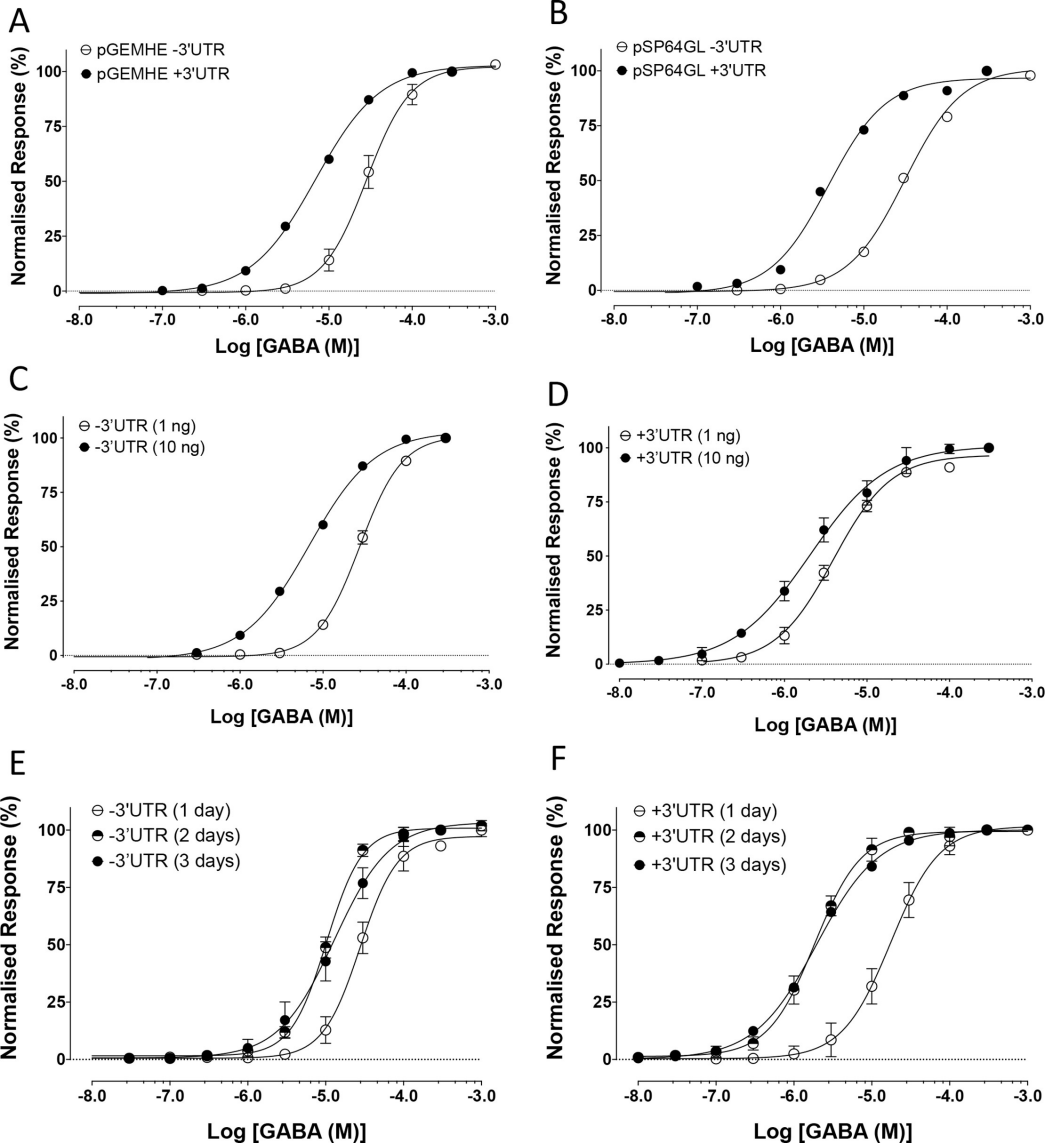
Changes in apparent affinity for GABA associated with differences in expression condition of BtRDL in Xenopus oocytes. A) GABA dose-responses curves from oocytes injected with cRNA synthesised from pGEMHE-BtRDL after digestion with *Xba*I to remove the 3’UTR (open circles) and from pGEMHE-BtRDL after digestion with *Nhe*I to retain the 3’UTR (filled circles). Oocytes were injected with 1 ng cRNA and recorded three-days post-injection. B) GABA dose-responses curves from oocytes injected with cRNA synthesised and pSP64GL-BtRDL after digestion with *Not*I to remove the 3’UTR (open circles) and from pSP64GL-BtRDL after digestion with *Bam*HI to retain the 3’UTR (filled circles) Oocytes were injected with 1 ng cRNA and recorded three-days post-injection. C) GABA dose-response curves from oocytes injected with either 1 ng cRNA (open circles) or 10 ng cRNA (filled circles) synthesised from pGEMHE-BtRDL after digestion with *Bam*HI to retain the 3’UTR and recorded three-days post-injection. D) GABA dose-response curves from oocytes injected with either 1 ng cRNA (open circles) or 10 ng cRNA (filled circles) synthesised from pSP64GL-BtRDL after digestion with *Xba*I to remove the 3’UTR and recorded three-days post-injection. E) GABA dose-response curves from oocytes recorded at one-day (open circles), two-days (half-filled circles) or three-days (filled circles) post-injection with cRNA (10 ng) synthesised from pSP64GL-BtRDL after digestion with *Bam*HI to retain the 3’UTR and recorded three-days post-injection. F) GABA dose-response curves from oocytes recorded at one-day (open circles), two-days (half-filled circles) or three-days (filled circles) post-injection with cRNA (10 ng) synthesised from pGEMHE-BtRDL after digestion with *Xba*I to remove the 3’UTR. In all cases, data are means ± SEM from at least three independent experiments.

Expression of BtRDL cRNA in Xenopus oocytes resulted in the formation of functional GABA-gated chloride channels. However, an unexpected observation was that receptor populations with significantly different sensitivities to GABA were detected when expressed from different batches of cRNA synthesised from the same plasmid construct (pGEMHE-BtRDL). This was the case, even when the same amount of cRNA was injected (1 ng) and when recordings were made at the same time post-injection (three-days). In all cases pGEMHE-BtRDL was linearised prior to *in vitro* cRNA synthesis by restriction enzyme digestion at a site downstream of the inserted RDL cDNA, but with restriction enzymes (*Xba*I or *Nhe*I) that cut at different locations (Fig 1B). Injection of cRNA (1 ng) synthesised after digestion of pGEMHE-BtRDL with *Nhe*I resulted in the expression of receptors with a GABA EC_50_ of 4.0 ± 1.1 μM (n = 3). In contrast, injection of the same quantity of cRNA synthesised after digestion of the plasmid with *Xba*I resulted in expression of receptors with a significantly lower apparent affinity for GABA (EC_50_ of 27.6 ± 1.6 μM, n = 3, p = 0.0003) (Fig 2A). In both cases, electrophysiological responses were recorded at three-days post-injection.

The only substantial difference between these two batches of cRNA (synthesised following plasmid digestion with either *Nhe*I or *Xba*I) was the presence or absence, respectively, of Xenopus β-globin 3’ UTR sequence (Fig 1B). During the original construction of expression vector pGEMHE, the rationale for including UTR sequence from Xenopus β-globin had been to increase levels of expression in Xenopus oocytes of inserted cDNAs [28]. However, this would not necessarily be expected to influence the apparent affinity for GABA. In an attempt to establish whether the presence or absence of the Xenopus β-globin 3’ UTR was responsible for the differences in apparent affinity, a similar experiment was performed with cRNA synthesised from an alternative plasmid construct (pSP64GL-BtRDL) in which BtRDL cDNA had been sub-cloned into pSP64GL (Fig 1B). As was the case with pGEMHE [28], pSP64GL was originally designed to optimise expression levels in Xenopus oocytes by the inclusion of the same Xenopus β-globin 3’ and 5’ UTRs [29, 30]. Injection of BtRDL cRNA synthesised from pSP64GL-BtRDL that either contained or lacked Xenopus β-globin 3’ UTR (after digestion of with *Bam*HI or *Not*I, respectively) had a similar influence on apparent affinity to that observed previously with cRNA synthesised from pGEMHE-BtRDL. Injection of pSP64GL-BtRDL cRNA (1 ng) containing the 3’ UTR generated receptors with a GABA EC_50_ of 3.7 ± 0.4 μM (n = 5). In contrast, injection of the same amount (1 ng) of pSP64GL-BtRDL cRNA lacking the 3’ UTR generated receptors with a significantly lower apparent affinity (EC_50_ of 31.4 ± 2.1 μM, n = 3, p < 0.0001) (Fig 2B). In all cases, electrophysiological responses were recorded at three-days post-injection.

### Influence of amount of cRNA on GABA EC_50_

To further examine the influence of expression conditions upon apparent affinity, additional experiments were performed in which the amount of cRNA injected was varied (Fig 2C & 2D). Injection of oocytes with 1 ng cRNA that lacked the 3’ UTR produced receptors with a GABA EC_50_ of 29.4 ± 1.0 μM (n = 6), whereas oocytes injected with 10 ng of the same cRNA produced a significantly lower GABA EC_50_ of 12.5 ± 1.0 μM (n = 8, p < 0.0001) (Fig 2C). In addition, a similar shift in EC_50_ was observed when oocytes were injected with cRNA that retained the 3’ UTR. Oocytes injected with 1 ng cRNA containing the 3’ UTR produced receptors with a GABA EC_50_ of 3.9 ± 0.6 μM (n = 8), whereas oocytes injected with 10 ng of the same cRNA produced a significantly lower GABA EC_50_ of 1.8 ± 0.4 μM (n = 9, p = 0.0095) (Fig 2D). In all cases, electrophysiological responses were recorded at three-days post-injection.

### Influence of length of time (post-injection) on GABA EC_50_

To examine the possible effect of incubation time (post-injection) on the apparent affinity for GABA, electrophysiological recordings were performed on oocytes at either one-day, two-days or three-days post-injection (Fig 2E & 2F). Oocytes injected with cRNA that lacked the 3’ UTR (10 ng) and examined at one-day post-injection produced receptors with a GABA EC_50_ of 27.3 ± 1.1 μM (n = 8), whereas oocytes examined two-days post-injection produced a significantly lower GABA EC_50_ of 10.8 ± 1.0 μM (n = 6, p < 0.0001). However, when oocytes were examined at three-days post-injection there was no further significant change in apparent affinity (GABA EC_50_ of 12.5 ± 1.0 μM (n = 8, p = 0.26) (Fig 2E). In addition, a similar shift in EC_50_ was observed when oocytes were injected with cRNA containing the 3’ UTR. Oocytes injected with cRNA containing the 3’ UTR (10 ng) examined one-day post-injection produced receptors with a GABA EC_50_ of 10.8 ± 1.2 μM (n = 6), whereas examined two-days post-injection produced a significantly lower GABA EC_50_ of 2.0 ± 0.7 μM (n = 8, p < 0.0001). In addition, as was found previously, when oocytes were examined at three-days post-injection there was no further significant change in apparent affinity (GABA EC_50_ of 1.8 ± 0.4 μM, n = 6, p = 0.80) (Fig 2F).

**Fig 3:**
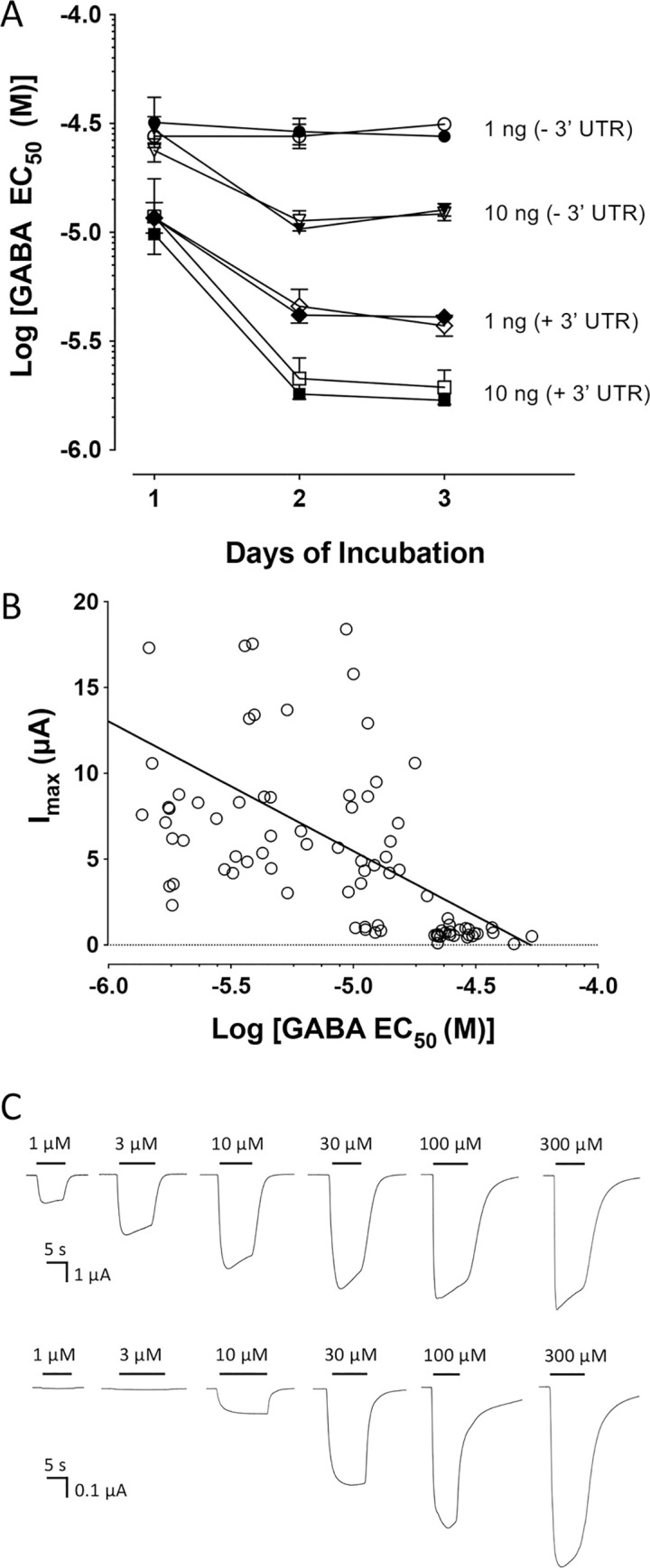
Changes in apparent affinity and current amplitude of BtRDL for GABA in oocytes under differing expression conditions. A) Apparent affinity for GABA is influenced by the amount of cRNA injected (1 ng or 10 ng), the presence or absence of a 3’ UTR (+3’UTR and -3’UTA), and the length of time post-injection before agonist responses were recorded (one- to three-days). Data are presented with 1 ng cRNA lacking a 3’ UTR (circles), 10 ng cRNA lacking a 3’ UTR (triangles), 1 ng cRNA containing a 3’ UTR (diamonds), 10 ng cRNA containing a 3’ UTR (squares). In all cases independent data were generated from cRNA synthesised from either pSP64GL (open symbols) and pGEMHE (filled symbol). B) Correlation between apparent affinity for GABA (EC_50_) and current amplitude (I_max_). Individual GABA EC_50_ values are plotted against the GABA I_max_, together with a line of best fit. Data are from 88 independently determined EC_50_ values from oocytes expressing BtRDL under a variety of expression conditions. This includes variations in the amount of cRNA (1 or 10 ng), the length of time post-injection (one-, two- or three-days) or the presence or absence of the 3’ UTR. C) Representative traces from oocytes expressing BtRDL, in response to application of a range of concentrations of GABA. Upper traces correspond to oocytes injected with 10 ng cRNA containing a 3’ UTR. Lower traces correspond to oocytes injected with 1 ng cRNA lacking a 3’ UTR. In both cases recordings were made three-days post-injection. Note: the vertical axis of the scale bars differs by a factor of 10.

Fig 3A and Table 1 provides a summary of EC_50_ data from a more extensive series of experiments involving changes in 1) the amount of cRNA injected, 2) the presence or absence of the 3’ UTR and 3) the length of time (post-injection) prior to recording (a total of 88 independently determined EC_50_ values). BtRDL receptors with the highest apparent affinity for GABA were produced by injecting 10 ng of cRNA containing a 3’ UTR and incubating for three-days (EC_50_ = 1.8 ± 0.4 μM; n = 9). In contrast, receptors with the lowest apparent affinity for GABA were produced by injecting 1 ng of cRNA lacking the 3’UTR and recording at one-day post-injection (EC_50_ = 29.7 ± 1.1 μM; n = 7). This corresponds to a 17-fold shift in apparent affinity.

**Table 1:** Heterologous expression conditions and agonist (GABA) apparent affinity (EC_50_).

Plasmid construct	Amount of	Day 1	Day 2	Day 3
(presence/absence 3’UTR)	cRNA (ng)	EC_50_ (μM)	EC_50_ (μM)	EC_50_ (μM)
BtRDL (-3’UTR)	1	29.7 ± 1.1 (n = 7)	28.3 ± 1.1 (n = 6)	29.4 ± 1.0 (n = 6)
BtRDL (-3’UTR)	10	27.3 ± 1.1 (n = 8)	10.8 ± 1.0 (n = 6)	12.5 ± 1.0 (n = 8)
BtRDL (+3’UTR)	1	11.6 ± 1.1 (n = 7)	4.4 ± 0.7 (n = 9)	3.9 ± 0.6 (n = 8)
BtRDL (+3’UTR)	10	10.8 ± 1.2 (n-6)	2.0 ± 0.7 (n = 8)	1.8 ± 0.4 (n = 9)

Data are means ± SEM.

### Changes in GABA EC_50_ and Imax

A consistent observation was that expression of BtRDL receptors with low apparent affinity (high EC_50_) were obtained from oocytes with relatively low maximum agonist responses (low I_max_), whereas receptors with high apparent affinity (low EC_50_) were obtained from oocytes with higher maximum agonist responses. The relationship between I_max_ and the EC_50_ for all expression conditions examined (88 independently determined EC_50_ values) is illustrated in Fig 3B. The correlation is strongest for receptors with the lowest apparent affinity (EC_50_ > 20 μM) which were all generated from oocytes with relatively small maximum responses (I_max_ < 1.5 μA). Representative traces from BtRDL expressed in two differing experimental conditions are illustrated in Fig 3C. For example, expression conditions that generated low EC_50_ values, such as injection of 1ng of BtRDL cRNA containing the 3’UTR, examined 1–3 days post-injection (EC_50_ = 11.6–3.9 μM; Table 1), consistently generated high maximum currents (I_max_ = 11.1 ± 0.9 μA; n = 24), whereas expression conditions that generated high EC_50_ values, such as injection of 1ng of BtRDL cRNA lacking the 3’UTR, examined 1–3 days post-injection (EC_50_ = 29.7–28.3 μM; Table 1) generated significantly lower maximum currents (I_max_ = 0.67 ± 0.04 μA; n = 19).

### Allosteric agonist activation of BtRDL

**Fig 4:**
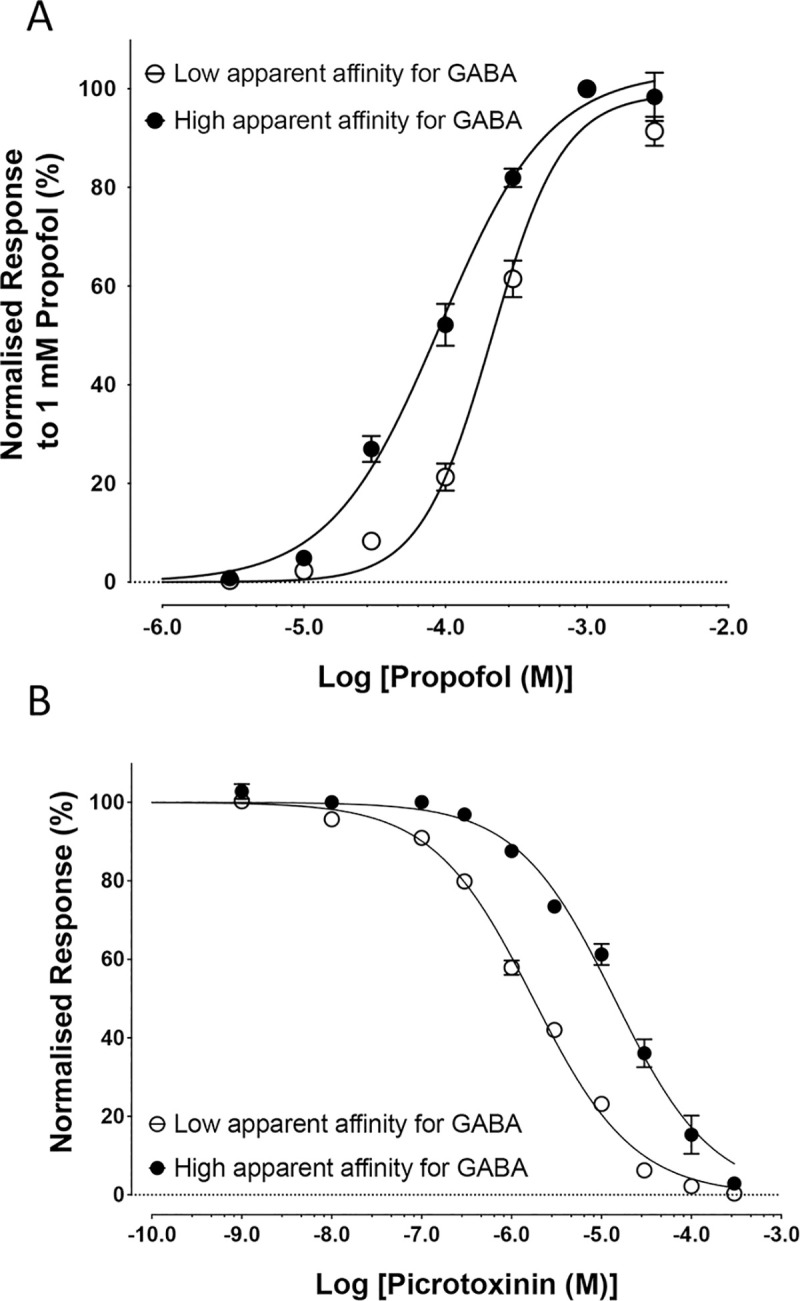
Changes in apparent affinity of BtRDL for propofol and picrotoxinin in oocytes under differing expression conditions. A) Propofol dose-response curves were generated from oocytes injected with cRNA synthesised from pSP64GL-BtRDL after digestion with *Bam*HI to retain the 3’UTR. Populations of BtRDL with low and high apparent affinity for GABA were generated by comparing oocytes injected with 1 ng cRNA, recorded one-day post-injection (filled circles) and 10 ng cRNA, recorded three-days post-injection (open circles). B) Picrotoxinin inhibition dose-response curves were generated from oocytes expressing BTRDL with either high or low apparent affinity for GABA (filled and open circles, respectively). BtRDL with high sensitivity was generated by injecting 10 ng cRNA synthesised from pGEMHE-BtRDL after digestion with *Nhe*I (to retain the 3’ UTR) and recorded three-days post-injection. BtRDL with low apparent affinity for GABA was generated by injecting 1 ng cRNA synthesised from pGEMHE-BtRDL after digestion with *Xba*I (to retain the 3’ UTR) and recorded three-days post-injection. In all cases, data are means ± SEM from at least five independent experiments.

Previous studies have reported that the anaesthetic propofol activates vertebrate GABA-gated chloride channels (GABA_A_ receptors) via an allosteric site [15, 16]. Here, we have shown that BtRDL can also be activated by propofol, producing dose-dependent responses that can be blocked by the antagonist picrotoxinin. In order to determine whether expression-dependent changes in apparent affinity of BtRDL affected an allosteric agonist (propofol), two populations of BtRDL were examined that had expression-dependent differences in apparent affinity for GABA. Although propofol displayed agonist activity on both BtRDL populations, the size of the propofol-evoked responses, relative to the maximal GABA response, were greater on BtRDL receptors that display high apparent affinity for GABA. Propofol yielded responses that were 37.8 ± 2.1% (n = 7) and 19.4 ± 3.2% (n = 6) of the maximal GABA response (300 μM) on BtRDL with high and low apparent affinity for GABA, respectively. In addition, Propofol had a significantly different apparent affinity (p < 0.0001) on the two receptor populations (82.9 ± 6.4 μM; n = 7 and 198.4 ± 19.1 μM; n = 6, respectively) (Fig 4A).

### Antagonist effects on BtRDL

To examine whether sensitivity to antagonists was altered in BtRDL populations with differing apparent affinity for GABA, studies were performed with picrotoxinin, a non-competitive antagonist of RDL receptors [9, 31]. Picrotoxinin caused a dose-dependent block of responses to an EC_50_ concentration of GABA (Fig 4B). However, sensitivity to antagonism by picrotoxinin was significantly different (p < 0.0001) on BtRDL populations that exhibited either high or low apparent affinity for GABA. On receptors with a high apparent affinity for GABA, responses to GABA were blocked by picrotoxinin with an IC_50_ of 14.0 ± 1.6 μM (n = 6). In contrast, picrotoxinin had a higher apparent affinity (IC_50_ = 1.7 ± 0.3 μM; n = 5) on receptors with a low apparent affinity for GABA. In both cases, the antagonist effect of picrotoxinin was examined on EC_50_ concentrations of GABA (1 μM and 30 μM for receptors with high and low apparent affinity for GABA, respectively).

### Co-expression of chaperone proteins

**Fig 5:**
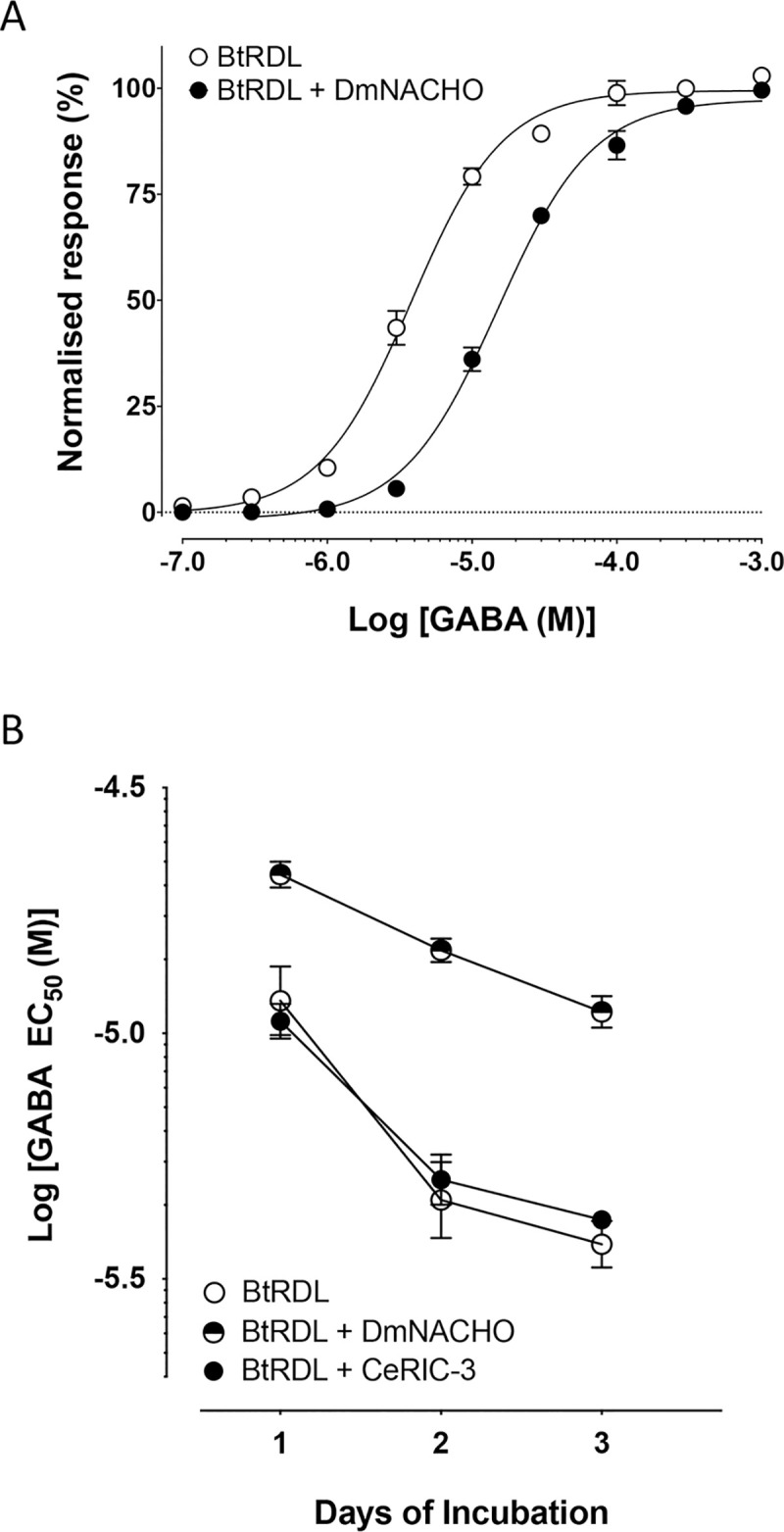
Influence of chaperone proteins DmNACHO and CeRIC-3 on apparent affinity of BtRDL. A) GABA dose-response curves were generated from oocytes injected with cRNA (1 ng) synthesised from pSP64GL-BtRDL after digestion with *Bam*HI to retain the 3’UTR, in the presence (filled circles) or absence (open circles) of cRNA (10 ng) encoding DmNACHO and recorded two-days post-injection. B) GABA EC_50_ values determined from oocytes injected with cRNA (1 ng) synthesised from pSP64GL-BtRDL after digestion with *Bam*HI to retain the 3’UTR and recorded as one-, two- or three-days post-injection. Data are presented for BtRDL expressed alone (open circles), co-expressed with DmNACHO (half-filled circles) and co-expressed with CeRIC-3 (filled circles). In all cases, data are means ± SEM from at least five independent experiments.

Previous studies have demonstrated that the efficiency of folding, assembly and cell-surface expression of pLGICs can be influenced by the co-expression of chaperone proteins [32]. For example, NACHO [23] and RIC-3 [33] are chaperone proteins that were originally identified as modulators of nAChRs. However, there is evidence that nAChR chaperones can enhance cell-surface expression of other classes of pLGIC [34] and, for this reason, co-expression studies were performed with BtRDL. Specifically, studies were performed with CeRIC-3, a previously described RIC-3 isoform isolated from *Caenorhabditis elegans* [22] and with NACHO cloned from Drosophila (DmNACHO). Co-expression of DmNACHO with BtRDL caused a significant reduction in apparent affinity for GABA (Fig 5A and Table 2). For example, at two-days post-injection, BtRDL expressed alone had a GABA EC_50_ of 4.6 ± 0.9 μM (n = 6) but had a significantly lower apparent affinity (EC_50_ = 14.8 ± 1.1 μM, n = 5, p = 0.0003) when co-expressed with DmNACHO (Fig 5A and Table 2) and was associated with a significantly reduced maximum current (I_max_ = 3.4 ± 0.6 μA when co-expressed with DmNACHO, compared with 10.8 ± 0.8 μA in the absence of DmNACHO, n = 15, p < 0.0001). Similar significant changes in EC_50_ were observed at one-day and three-days post-injection (Fig 5B, Table 2). In contrast, co expression with CeRIC-3 had no significant effect on agonist sensitivity of BtRDL (Fig 5B and Table 2).

**Table 2:** Influence on co-expressed chaperones on GABA apparent affinity (EC_50_).

	Day 1	Day 2	Day 3
	EC_50_ (μM)	EC_50_ (μM)	EC_50_ (μM)
BtRDL	11.6 ± 2.0 (n = 4)	4.6 ± 0.9 (n = 6)	3.7 ± 0.4 (n = 5)
BtRDL + CeRIC-3	10.6 ± 1.1 (n = 5)	5.0 ± 0.9 (n = 5)	4.2 ± 1.0 (n = 5)
BtRDL + DmNACHO	21.0 ± 1.1 (n = 5)	14.8 ± 1.1 (n = 5)	11.1 ± 1.1 (n = 5)

BtRDL was expressed by injection of 1 ng cRNA (+3’UTR) in the presence or absence of 10 ng cRNA encoding CeRIC-3 or DmNACHO. Data are means ± SEM.

### Species selectivity

**Fig 6:**
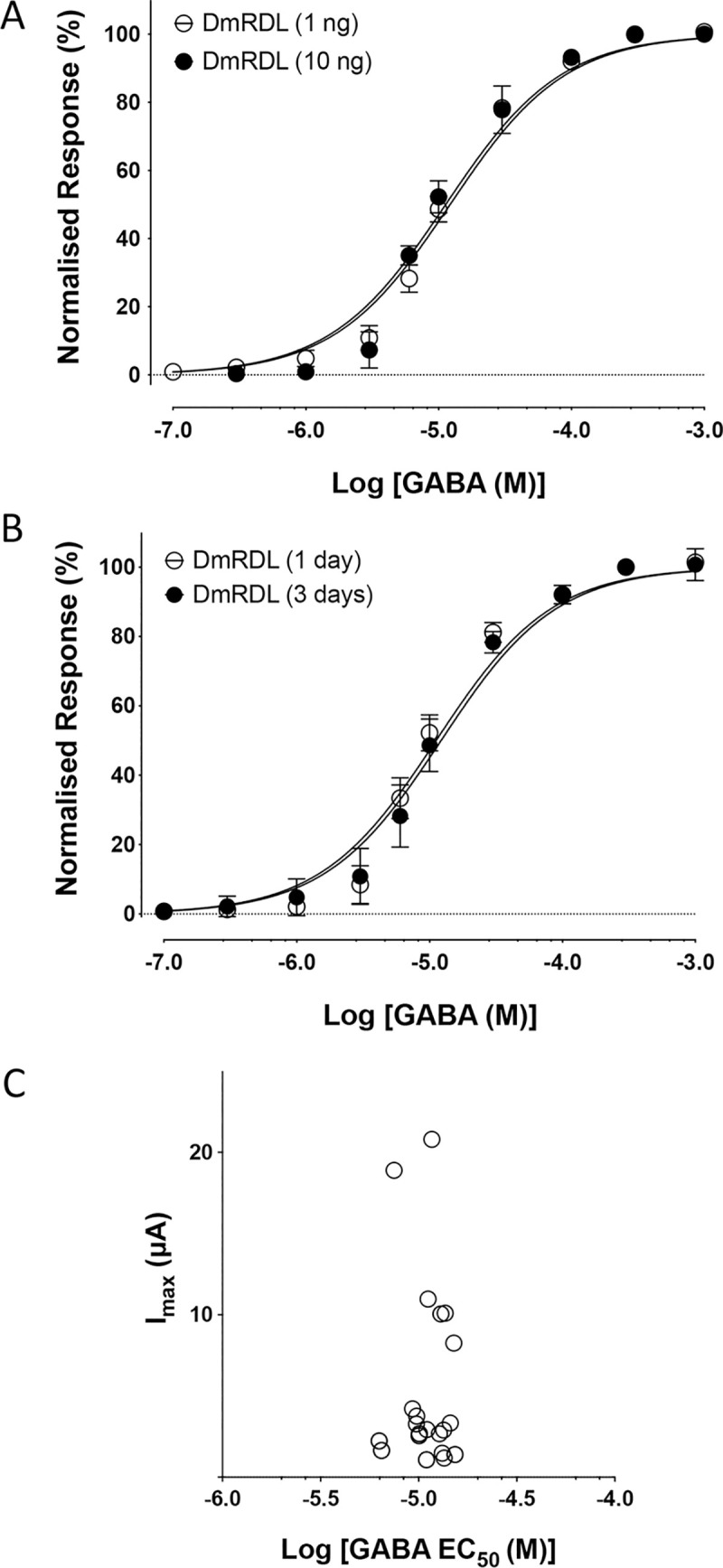
Absence of changes in apparent affinity for GABA in oocytes expressing DmRDL under differing expression conditions. A) GABA dose-response curves from oocytes injected with either 1 ng cRNA (open circles) or 10 ng cRNA (filled circles) synthesised from pGEMHE-BtRDL and recorded two-days post-injection. B) GABA dose-response curves from oocytes injected with cRNA (1 ng) synthesised from pGEMHE-BtRDL and recorded at either one-day post-injection (open circles) or three-days post-injection (filled circles). In all cases, data are means ± SEM from at least five independent experiments. C) Individual GABA EC_50_ values are plotted against the GABA I_max_. Data are from 28 independently determined EC_50_ values from oocytes expressing BtRDL under a variety of expression conditions.

In contrast to our findings with BtRDL, no significant difference in apparent affinity for GABA was observed when DmRDL was expressed in Xenopus oocytes under a similar range of expression conditions (Fig 6). For example, EC_50_ values were not significantly different when oocytes were injected with either 1 ng cRNA (11.2 ± 1.1 μM; n = 5) or 10 ng cRNA (10.5 ± 1.1 μM; n = 5) and recorded two-days post-injection (p = 0.27) (Fig 6A). Similarly, EC_50_ values were not significantly different when oocytes that had been injected with the same amount of cRNA (1 ng) were recorded after either one-day (11.6 ± 1.1 μM; n = 7) or three-days (12.2 ± 0.9 μM, n = 5, p = 0.70) (Fig 6B). In contrast to the findings with BtRDL (Fig 3), there was no correlation between EC_50_ and I_max_ for DmRDL (Fig 6C).

## Discussion

Following the molecular cloning and functional expression of BtRDL, one of our initial observations was that the apparent affinity (EC_50_) for GABA in Xenopus oocytes was influenced by the batch of cRNA that was used for heterologous expression. More extensive studies, including those with BtRDL sub-cloned into two different plasmid expression vectors (pGEMHE and pSP64GL), indicated that the differences in apparent affinity could be attributed to the presence or absence of Xenopus β-globin 3’ UTR sequence in the transcribed cRNA. It is well established that 3’ UTRs can influence mRNA stability and translation efficiency [35]. Indeed, this was the rationale behind the incorporation of Xenopus β-globin UTR sequences into these plasmid expression vectors, both of which were originally designed to optimise heterologous expression of cloned genes in Xenopus oocytes [28–30].

One of the central findings of the present study is that BtRDL had low apparent affinity for GABA (high EC_50_) under expression conditions generating low maximum agonist responses (low I_max_), whereas receptors with high apparent affinity (low EC_50_) were obtained from oocytes with higher maximum agonist responses. A possible explanation for these effects is that properties such as apparent affinity are influence by changes in conformation resulting from differences in receptor density. When expressed in Xenopus oocytes, the apparent affinity for GABA was influenced by 1) the presence or absence of a 3’ UTR, 2) the amount of cRNA injected and 3) incubation time prior to functional analysis. The apparent affinity was also influenced by co-expression of a chaperone protein (NACHO). In addition, expression-dependent changes in apparent affinity were observed with an allosteric agonist (propofol) and a non-competitive antagonist (picrotoxinin).

Insect RDL receptors form homomeric GABA-gated chloride channels but are members of a diverse family of pLGICs that contains both homomeric and heteromeric receptors [1, 2]. Previous studies have reported differences in agonist apparent affinity of heteromeric pLGICs and such effects have been attributed to differences in subunit stoichiometry [36, 37]. However, expression-dependent changes in apparent affinity, similar to those reported in the present study with BtRDL, have also been reported previously for homomeric receptors. This includes vertebrate homomeric glycine receptors [38–41], which are closely related members of the pLGIC family. In addition, such expression-dependent changes have been reported with structurally unrelated receptors, such as the ATP-gated P2X_2_ receptor [42] and the transient receptor potential TRPV1 channel [43]. In these previously studies with homomeric receptors, it has been suggested that expression-dependent changes in apparent affinities may be a consequence of density-dependent changes in inter-receptor cooperativity or due to altered interactions with intracellular factors, both of which are likely to be associated with changes in receptor conformation [38, 40, 42, 43]. Our present findings are consistent with this possibility. In addition to evidence for expression-dependent changes with receptors expressed in Xenopus oocytes (as reported here), such effects have also been reported for receptors expressed in other expressions systems, such as cultured mammalian cell lines [39, 41].

The influence of expression conditions upon apparent affinity for GABA observed with BtRDL are perhaps illustrated most clearly in Fig 3A. The lowest apparent affinity for GABA was observed when oocytes were injected with low amounts of cRNA (1 ng) that lacked a 3’ UTR (Fig 3A; Table 1). Under such expression conditions, receptors with high apparent affinity were never observed, even at three-days post-injection (Fig 3A; Table 1), perhaps because a sufficiently high receptor density was never achieved. In contrast, the highest apparent affinity was observed when oocytes were injected with high amounts of cRNA (10 ng) that contained a 3’ UTR (Fig 3A; Table 1). In such conditions, receptors with higher apparent affinity were observed at the earliest time point examined (one-day) and increase significantly over time, perhaps due to a further increase in receptor density. Also illustrated in Fig 3A and summarised in Table 1 is evidence that, in addition to receptors with low and high apparent affinities for GABA (~2 μM and ~ 30 μM, respectively), BtRDL populations were also detected with intermediate apparent affinities (~ 4 μM and ~12 μM) under certain expression conditions. Although it is not possible to be certain whether such changes are associated with differences in either binding or gating, this would seem to suggest that BtRDL is able to adopt a variety agonist-bound conformations with differing agonist affinities as a consequence of altered expression conditions.

It is of interest that, in contrast to our findings with BtRDL, no evidence for expression-dependent changes in apparent affinity were observed with DmRDL. The reason for this species-selectivity is unclear but can presumably be accounted for by differences in their amino acid sequence. The two proteins differ to the greatest extent in their N-terminal signal sequences and within the intracellular (M2-M3) loop domains but otherwise have a high degree of sequence similarity (Fig 1A). One approach that might be useful in investigating the role of individual amino acids or protein domains would be the use of site-directed mutagenesis or the generation of artificial cDNA chimeras.

It is notable that changes in apparent affinity were not restricted to ligands interacting with the extracellular orthosteric agonist binding site. Changes in the apparent affinity were observed both for activation by the allosteric ligand propofol and with the non-competitive antagonist picrotoxinin, both of which are thought to interact with transmembrane binding sites [16, 44]. These findings with propofol and picrotoxinin suggest that the changes associated with different expression conditions may be a consequence of relatively widespread changes in protein conformation which, in turn may be a consequence of receptor density. It is interesting that, whereas changes in expression conditions that would be expected to lead to higher receptor density were associated with increases in the apparent affinity for the two agonists examined (GABA and propofol), the same changes were associated with lower apparent affinity for the antagonist picrotoxinin. A possible explanation for this may be that the effects on ligands interacting at different binding sites are influenced differently by global changes in receptor conformation associated with different expression conditions.

Due to evidence indicating that changes in the apparent affinity of BtRDL were associated with changes in level of receptor expression, we aimed to examine whether similar effects might be observed by the co-expression of chaperone proteins. RIC-3 is a chaperone that was originally cloned from *C*. *elegans* [33] and has been reported to enhance the assembly, maturation and cell-surface expression of a variety of pLGICs [32, 34]. The human chaperone NACHO, a small (167 amino acid) protein with four putative transmembrane domains, was originally cloned in a high-throughput assay aimed at identifying proteins able to facilitate functional expression of the human α7 nAChR [23]. Drosophila NACHO (DmNACHO), examined in this study, is a closely related 160 amino acid protein that has also been reported to facilitate functional expression of human α7 nAChR [23]. Whereas co-expression of CeRIC-3 with BtRDL had no apparent effect on the apparent affinity for GABA, co-expression of DmNACHO resulted in expression of BtRDL with a lower apparent affinity for GABA. This might indicate that DmNACHO is able to interact with BtRDL, as it does with the α7 nAChR, but perhaps disrupts rather than enhances folding and assembly of BtRDL. However, further work will be required to identify the mechanism by which proteins such as NACHO are able to influence assembly and folding of pLGICs.

Our evidence for a change in the apparent affinity for GABA with BtRDL and a correlation between apparent affinity and levels of expression is similar to what has been reported previously with vertebrate glycine-gated ion channels. For example, studies with the human GlyR subunits in Xenopus oocytes reported that at high levels of expression were associated with the appearance of a population of receptors with higher apparent affinity for glycine [38, 40]. In addition, it was reported that the apparent affinities of partial agonists on GlyRs were also correlated with expression levels [40]. Interestingly, whereas we have found that the apparent affinity of the non-competitive antagonist picrotoxinin was reduced on BtRDL that had high apparent affinity for GABA, previous studies with GlyR α1 reported no change in apparent affinity for picrotoxin [38]. The same study did, however, observe an expression-depended reduction in apparent affinity for the competitive antagonist strychnine in populations of GlyR that had high apparent affinity for glycine [38]. It seems probable that all such effects can be explained by global expression-dependent changes in protein conformation but, at present, the precise mechanisms by which this occurs remains unknown.

In previous studies with GlyRs, it has been suggested that expression-dependent changes in apparent affinity may be a consequence of enhanced inter-receptor interactions resulting from high density packing [40]. Indeed, the possibility of inter-molecular cooperativity between transmembrane proteins is an idea that has a long history [45]. With regard to the expression-dependent effects observed with GlyRs, it has been suggested that high-density packing of individual receptors into larger supramolecular structures could cause differences in receptor properties during agonist activation, due to lateral allosteric coupling between single channel [38]. This seems to be a plausible hypothesis and would be consistent with the observations we have reported in the present study with BtRDL.
